# The insulin receptor regulates the persistence of mechanical nociceptive sensitization in flies and mice

**DOI:** 10.1242/bio.059864

**Published:** 2023-06-01

**Authors:** Yan Wang, Roger Lopez-Bellido, Xiaojiao Huo, Annemieke Kavelaars, Michael J. Galko

**Affiliations:** ^1^ Department of Genetics, University of Texas MD Anderson Cancer Center, Houston, TX 77030, USA; ^2^ Department of Symptom Research, University of Texas MD Anderson Cancer Center, Houston, TX 77030, USA; ^3^ Neuroscience Graduate Program, The MD Anderson Cancer Center UTHealth Graduate School of Biomedical Sciences, Houston, TX 77030, USA; ^4^ Genetics & Epigenetics Graduate Program, The MD Anderson Cancer Center UTHealth Graduate School of Biomedical Sciences, Houston, TX 77030, USA

**Keywords:** Nociception, Mechanical sensitization, Insulin receptor, Mouse, *Drosophila*

## Abstract

Early phase diabetes is often accompanied by pain sensitization. In *Drosophila*, the insulin receptor (*InR*) regulates the persistence of injury-induced thermal nociceptive sensitization. Whether *Drosophila InR* also regulates the persistence of mechanical nociceptive sensitization remains unclear. Mice with a sensory neuron deletion of the insulin receptor (*Insr*) show normal nociceptive baselines; however, it is uncertain whether deletion of *Insr* in nociceptive sensory neurons leads to persistent nociceptive hypersensitivity. In this study, we used fly and mouse nociceptive sensitization models to address these questions. In flies, *InR* mutants and larvae with sensory neuron-specific expression of RNAi transgenes targeting *InR* exhibited persistent mechanical hypersensitivity. Mice with a specific deletion of the *Insr* gene in Nav1.8^+^ nociceptive sensory neurons showed nociceptive thermal and mechanical baselines similar to controls. In an inflammatory paradigm, however, these mutant mice showed persistent mechanical (but not thermal) hypersensitivity, particularly in female mice. Mice with the Nav1.8^+^ sensory neuron-specific deletion of *Insr* did not show metabolic abnormalities typical of a defect in systemic insulin signaling. Our results show that some aspects of the regulation of nociceptive hypersensitivity by the insulin receptor are shared between flies and mice and that this regulation is likely independent of metabolic effects.

## Teaser

**Summary:** This paper demonstrates a conserved role for the insulin receptor in regulating the persistence of injury-induced mechanical nociception in flies and mice.

## INTRODUCTION

The fruit fly (*Drosophila melanogaster*) has emerged over the last decade as a powerful system to genetically dissect nociception and nociceptive sensitization (Im and Galko, 2012; Milinkeviciute et al., 2012). Early work established that *Drosophila* avoid noxious stimuli through conserved transient receptor potential (TRP) channels (Hwang et al., 2012; Kang et al., 2010; Tracey et al., 2003). These avoidance responses, such as the aversive rolling of *Drosophila* larvae, are enabled by multidendritic peripheral sensory neurons (Gao et al., 1999; Hwang et al., 2007) whose elaborate dendritic arbors tile over the barrier epidermis. These neurons are the functional counterparts of unmyelinated nociceptors in vertebrates. Nociceptive responses in *Drosophila* include detection of noxious heat (Babcock et al., 2009; Tracey et al., 2003), cold (Turner et al., 2016), mechanical (Kim et al., 2012; Lopez-Bellido et al., 2019b; Mauthner et al., 2014), and chemical (Lopez-Bellido et al., 2019a) stimuli. There is additional complexity to the response, however. As in vertebrates (Gold and Gebhart, 2010), tissue injury is capable of causing transient hypersensitivity to both noxious (hyperalgesia) and non-noxious (allodynia) stimuli. Some of the molecular pathways that mediate these responses such as tumor necrosis factor (TNF) signaling (Babcock et al., 2009; Cunha et al., 1992, 2005), Substance P/Tachykinin (Im et al., 2015; Laird et al., 2001; Weng et al., 2001), and Hedgehog signaling (Babcock et al., 2011; Liu et al., 2018) are conserved. Finally, flies possess second-order and higher order central interneurons (Hu et al., 2017; Ohyama et al., 2015; Yoshino et al., 2017) that comprise a nociceptive circuit and confer the potential for nociceptive responses to be modulated by competing sensory inputs.

Flies have also proven to be a useful model of insulin-like signaling (ILS). *Drosophila* possess a clear insulin receptor ortholog, *InR* (Fernandez et al., 1995), insulin-like peptides (Rulifson et al., 2002), and conserved downstream pathway components (Clancy et al., 2001; Goberdhan et al., 1999). Disruption of ILS through mutation or *in vivo* RNAi-mediated knockdown leads to a wide variety of phenotypes including increased longevity (Tatar et al., 2001), decreased cell size (Böhni et al., 1999; Rulifson et al., 2002), and metabolic effects that mirror aspects of diabetes in vertebrates (Musselman et al., 2011). A prior study from our lab indicated that *Drosophila InR* is required specifically in nociceptive sensory neurons for the resolution of injury-induced thermal nociceptive sensitization (Im et al., 2018). In this same study (Im et al., 2018), *Drosophila* larvae mimicking type I diabetes (Rulifson et al., 2002) or type II diabetes (reared on a high sugar diet) (Musselman et al., 2011) also exhibited persistent injury-induced nociceptive hypersensitivity, suggesting a potential connection between sensory neuron loss of ILS and hypersensitivity. Here, we test whether fly *InR* is similarly required for the persistence of mechanical nociceptive hypersensitivity.

In mice, homozygous *Insr* mutants are lethal (Accili et al., 1996). Tissue specific deletion of *Insr* combining a floxed allele (Bruning et al., 1998) with a Nestin-Cre driver that expresses in neuronal and glial precursors led to no obvious sensory phenotypes (Brüning et al., 2000). As with most tissues, *Insr* is reported to be expressed in sensory neurons (Sugimoto et al., 2002, 2000). A more specific deletion of *Insr* in sensory neurons using *Advillin-Cre* (Zurborg et al., 2011), revealed no obvious alterations to baseline nociceptive sensitivity in the absence of injury (Grote et al., 2018). Nociceptive hypersensitivity to inflammatory injury was not examined in this study, but systemic impacts on sugar metabolism (elevated serum insulin and glucose intolerance) were observed (Grote et al., 2018). Here, motivated in part by our prior studies in *Drosophila*, we tested whether deletion of *Insr* in Nav1.8-positive nociceptive sensory neurons (Agarwal et al., 2004) regulates the onset/duration of either thermal or mechanical hypersensitivity. We also measured whether such neuron-specific deletion impacts systemic measures of metabolism.

## RESULTS

### *Drosophila InR* regulates the persistence of mechanical nociceptive sensitization

**Fig. 1.**
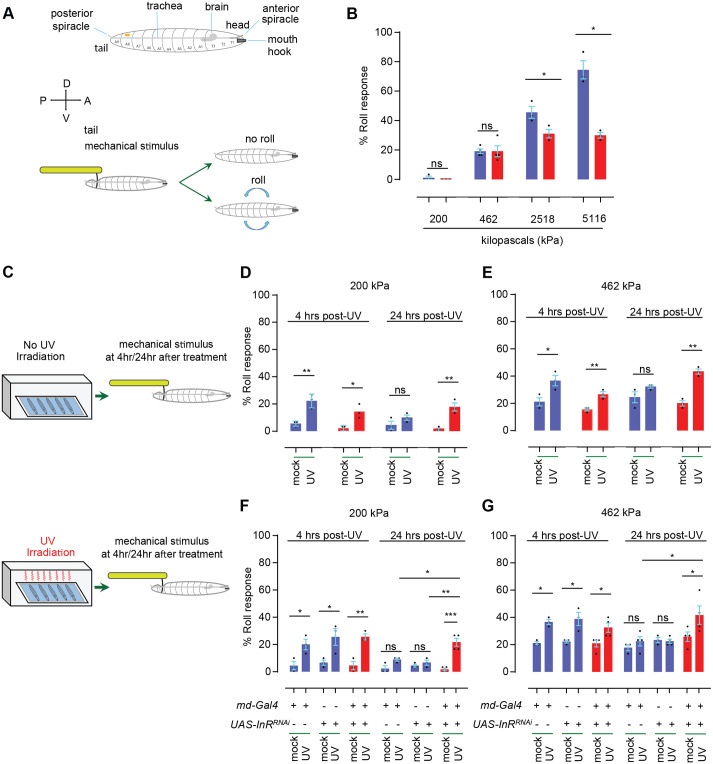
**Mechanical hypersensitivity in flies lacking *InR*.** (A) Schematic of mechanical nociception assay. Third instar *Drosophila* larvae (prominent anatomical features noted) are poked with a custom-designed and built larval von Frey filament (see Materials and Methods) exerting a defined amount of pressure. The resulting behavior is recorded. Sub-threshold pressures do not result in aversive rolling, while higher noxious pressures increase the percentage of larvae that perform the rolling behavior. (B) Behavioral dose-response curve of control (*w^1118^*, blue bar) and *InR* mutant larvae (*InR^e19^/InR^93Dj4^*, red bar). (C) Schematic of mechanical hypersensitivity assay. Larvae are either mock-irradiated (control) or UV-irradiated to induce epidermal tissue damage. At the indicated times post-irradiation, larvae were behaviorally tested using the mechanical nociception assay described in A. (D,E) Quantitation of mechanical allodynia (D, mostly sub-threshold 200 kPa probe) and mechanical hyperalgesia (E, mildly noxious 462 kPa probe) in control (blue bar) and *InR* mutant larvae (red bar). (F,G) Quantitation of mechanical allodynia (F, mostly sub-threshold 200 kPa probe) and mechanical hyperalgesia (G, mildly noxious 462 kPa probe) in larvae with various genetic controls (*md-Gal4* alone, *UAS-InR^RNAi^* alone, blue bars) or in larvae with *InR* targeted by *UAS-InR^RNAi^* in nociceptive sensory neurons (*md-Gal4* driver). Red bars indicate experimental larvae harboring both components of the Gal4/UAS system and thus expressing *UAS-InR^RNAi^* within nociceptive sensory neurons. For the quantitation panels (B, D-G), mean±s.e.m., *n*=3 or 4 sets of 30 larvae per condition. Black dot, a set of 30 larvae. Two-tailed unpaired Student's *t*-test was used for panels B, D and E; one-way ANOVA (followed by Tukey post hoc test) was used for panels F and G. ns, not significant, *, *P*<0.05, **, *P*<0.01, ***, *P*<0.001.

We first set to test whether the persistent thermal nociceptive sensitization observed in flies with *InR* mutation and with sensory neuron-specific expression of *UAS-InR^RNAi^* transgenes (Im et al., 2018) extends to mechanical nociception. To do this, we used a newly refined assay for mechanical nociception (Lopez-Bellido and Galko, 2020; Lopez-Bellido et al., 2019b) (see schematic, Fig. 1A). In the absence of injury, control larvae showed a normal dose-response curve to sub-noxious (≤ 200 kPa) and noxious (> 200 kPa) mechanical stimuli (blue bars, Fig. 1B). By contrast, larvae transheterozygous for a viable combination of *InR* alleles (Im et al., 2018; Tatar et al., 2001) showed normal mechanical responsiveness at the low end of the noxious range (462 kPa or below) but increasingly impaired responsiveness at higher pressures (red bars, Fig. 1B).

Based on these baseline data we decided to use a 200 kPa probe (almost no aversive rolling in controls and mutants) to test for onset and persistence of injury-induced mechanical allodynia and a 462 kPa probe (∼20% aversive rolling in controls and mutants) to test for induction and persistence of injury-induced mechanical hyperalgesia (see schematic, Fig. 1C). Previously published experiments established that 4 h after UV-irradiation is appropriate to test for onset of acute mechanical hypersensitivity and 24 h is suitable to test for persistence and/or resolution of this acute response (Lopez-Bellido and Galko, 2020). Although there were slight, non-significant variations in baseline responsiveness without UV irradiation (probably a consequence of *InR* mutant larvae being slightly smaller than controls), both control larvae and *InR* transheterozygotes showed newly acquired responsiveness (mechanical allodynia) to the 200 kPa probe and increased responsiveness (mechanical hyperalgesia) to the 462 kPa probe (Fig. 1D,E). In control larvae, this transient hypersensitivity had resolved close to baseline responsiveness by 24 h post-irradiation (Fig. 1D,E). By contrast, *InR* transheterozygotes exhibited persistent mechanical allodynia and hyperalgesia- they did not return back to baseline (Fig. 1D,E).

Finally, we tested whether *InR* function in mechanical allodynia is localized to peripheral multidendritic neurons, which are responsible for detecting noxious mechanical stimuli (Hwang et al., 2007; Kim et al., 2012; Lopez-Bellido et al., 2019b). Control larvae bearing only a Gal4 driver targeting nociceptive multidendritic sensory neurons (*md-Gal4*) (Gao et al., 1999) or the *UAS-InR^RNAi^* transgene (Ni et al., 2011) exhibited normal mechanical baselines and normal sensitization following UV-induced tissue injury (Fig. 1F,G). Larvae bearing both transgenes (and thus expressing *UAS-InR^RNAi^* in multidendritic nociceptive sensory neurons) also exhibited a normal baseline (∼0% responders to 200 kPa and ∼20% responders to 462 kPa) in the absence of injury and a normal peak of sensitization 4 h post-injury (Fig. 1F,G). This sensitization persisted in these larvae beyond the normal duration; the acute sensitization response did not resolve back to baseline at 24 h post-injury (Fig. 1F,G). These data indicate that in fly larvae *InR* is required for optimal baseline sensitivity to noxious mechanical stimuli and, similar to what has been observed for thermal sensitization (Im et al., 2018), also regulates the persistence of mechanical sensitization to both sub-noxious and mildly noxioius mechanical pressures.

### Sensory neuron specific deletion of mouse *Insr*

**Fig. 2.**
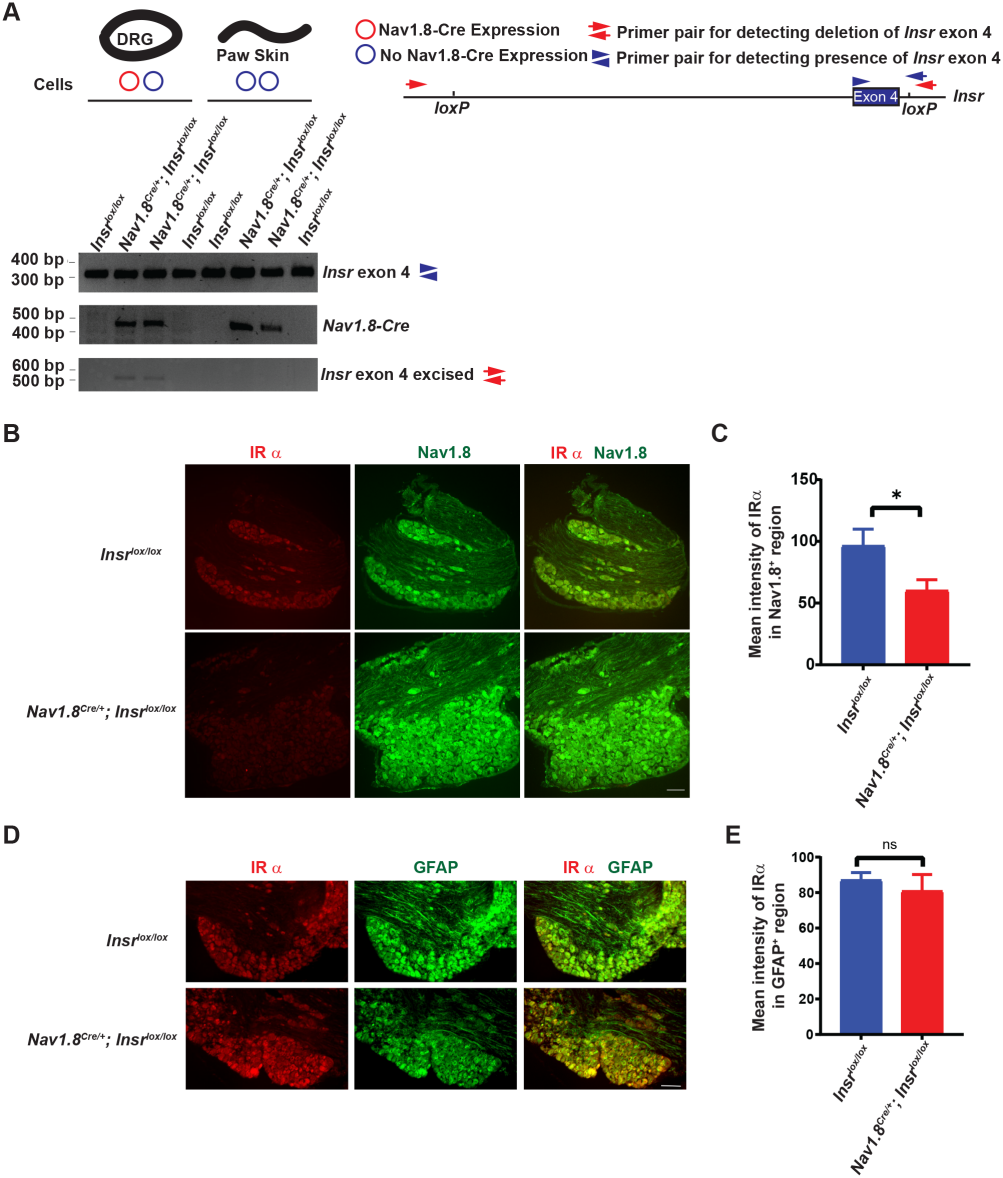
**Sensory neuron specific deletion of mouse *Insr*.** (A) Confirmation of Cre recombinase-mediated excision of insulin receptor exon 4 flanked by *loxP* sites, partial map of *Insr* locus to show primer pair locations. Genomic DNA as template from DRGs (thick black circle) or paw skin (black wavy line) from mice of the indicated genotypes was PCR-amplified using primers suitable for detecting the unrecombined floxed *Insr* exon 4 (top row, primers=blue arrowheads), Cre recombinase (middle row, primers not shown), and deleted *Insr* exon 4 (bottom row, primers=red arrows). (B) Cryosections of DRG tissue dissected from *Insr^lox/lox^* and *Nav1.8^Cre/+^;Insr^lox/lox^* mice immunostained for insulin receptor alpha subunit (IRα, red) and Nav1.8 (green). Scale bar: 100 µm. (C). Quantification of the intensity of IRα staining in Nav1.8^+^ DRG region of *Insr^lox/lox^* and *Nav1.8^Cre/+^;Insr^lox/lox^* mice. *Insr^lox/lox^*, *n*=10 DRGs from five mice (two females and three males). *Nav1.8^Cre/+^;Insr^lox/lox^*, *n*=9 DRGs from five mice (two females and three males). Mean±s.e.m. Two-tailed unpaired Student's *t*-test was used for statistical analysis. *, *P*<0.05. (D) Cryosections of DRG tissue dissected from *Insr^lox/lox^* and *Nav1.8^Cre/+^;Insr^lox/lox^* mice immunostained for IRα (red) and GFAP (green). Scale bar: 100 µm. (E). Quantitation of the intensity of IRα staining in GFAP^+^ DRG region of *Insr^lox/lox^* and *Nav1.8^Cre/+^;Insr^lox/lox^* mice. *Insr^lox/lox^*, *n*=10 DRGs from five mice (two females and three males). *Nav1.8^Cre/+^;Insr^lox/lox^*, *n*=9 DRGs from five mice (two females and three males). Mean±s.e.m. Two-tailed unpaired Student's *t*-test was used for statistical analysis. ns, not significant.

We wanted to test whether the abnormally persistent nociceptive sensitization observed in fly *InR* mutants might also be observed in a parallel experiment in mice. To do this, we bred mice heterozygous for a Cre driver (*Nav1.8-Cre*) specific for nociceptive sensory neurons (Agarwal et al., 2004) and homozygous for a floxed allele of *Insr* (Bruning et al., 1998), the locus that encodes the mouse insulin receptor. Using PCR primers specific for the intact floxed allele we detected an appropriately sized DNA band/product in all mice bearing the allele (Fig. 2A). Only mice bearing the *Nav1.8-Cre* driver generated a PCR band specific for the *Cre* gene (also Fig. 2A). Importantly, deletion of exon 4 of the *Insr* locus was tissue-specific- DNA harvested from DRG which contains the cell bodies of nociceptive sensory neurons, showed a PCR product specific for the excised locus when the Cre protein was present (Fig. 2A). As expected, such a band was absent when skin tissue was used as the DNA source, whether or not the Cre transgene was present (Fig. 2A). These data indicated that combining the *Nav1.8-Cre* transgene with the floxed allele of *Insr* leads to a tissue-specific deletion of a portion of the mouse *Insr* locus.

We also examined insulin receptor protein levels in the DRG of mice harboring *Insr^lox/lox^* with or without the Cre driver (Fig. 2B). We performed double immunofluorescence analysis with an antibody that recognizes Nav1.8, to mark cells that should be expressing the Cre driver, and with an antibody that recognizes the alpha chain of the mouse insulin receptor (IRα, Fig. 2B). Quantitation of IRα levels revealed a reduction in the level of Insr in Nav1.8^+^ cells in mice harboring the Cre driver but not in littermate controls harboring only the *Insr^lox/lox^* allele (Fig. 2C). We also performed double immunofluorescence analysis on the DRGs with an antibody that recognizes glial fibrillary acidic protein (GFAP), to label glial cells that should not be expressing the Cre driver, and with the antibody that recognizes the alpha chain of the mouse insulin receptor (Fig. 2D). Quantitation of IRα levels revealed no significant difference in the level of insulin receptor protein in GFAP^+^ cells between mice harboring the Cre driver and sibling controls with only the *Insr^lox/lox^* allele (Fig. 2E). These data reveal that in addition to the DNA deletion at the *Insr* locus there is a reduction in IRα protein levels specifically in targeted Nav1.8^+^ sensory neurons.

### Mouse *Insr* regulates the persistence of inflammatory mechanical hypersensitivity in females

**Fig. 3.**
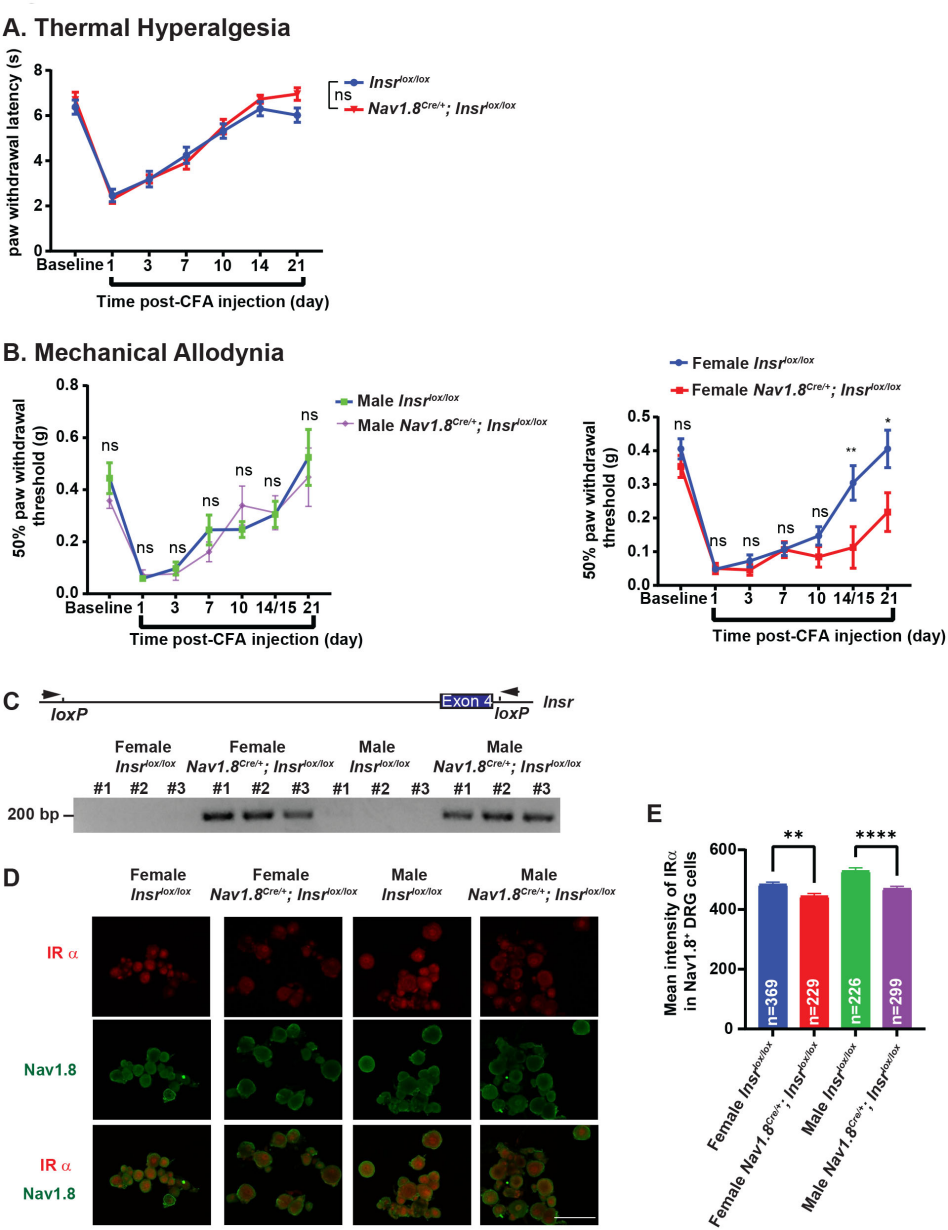
**Behavioral analysis of mice lacking sensory neuron-specific *Insr*.** (A) Quantitation of baseline thermal nociception and thermal hyperalgesia after CFA-induced local inflammation in *Insr^lox/lox^* controls and *Nav1.8^Cre/+^;Insr^lox/lox^* mice. *Insr^lox/lox^*, *n*=8 (four females and four males); *Nav1.8^Cre/+^;Insr^lox/lox^*, *n*=8 (four females and four males). Mean±s.e.m. Two-way repeated measures ANOVA with Geisser-Greenhouse correction followed by Sidak's multiple comparison test was used for statistical analysis. ns, not significant. (B) Graph shows 50% paw withdrawal threshold of *Insr^lox/lox^* and *Nav1.8^Cre/+^;Insr^lox/lox^* mice with the mice separated by sex. Male *Insr^lox/lox^*, *n*=5; male *Nav1.8^Cre/+^;Insr^lox/lox^*, *n*=6; female *Insr^lox/lox^*, *n*=19; female *Nav1.8^Cre/+^;Insr^lox/lox^*, *n*=13. *Nav1.8^Cre/+^;Insr^lox/lox^* mice of both sexes did not show defects in baseline mechanical nociception but female mice of this genotype exhibited a slow recovery in mechanical allodynia at 14/15 days after CFA-induced local inflammation. Mean±s.e.m. Based on normality test, two-tailed unpaired *t*-test was used for statistical analysis of female baseline, day 7 and day 21, and male baseline, day 3, day 7, day 14, and day 21 data, and two-tailed unpaired Mann–Whitney test was used for statistical analysis of female day 1, day 3, day 10, day 14, and male day 1 and day 10 data. ns, not significant. *, *P*<0.05, **, *P*<0.01. (C) Analysis of Cre recombinase-mediated excision of insulin receptor exon 4 flanked by *loxP* sites using primers suitable for detecting the recombined region (resulting in an ∼200 bp band). Genomic template DNA from DRGs from male or female mice of the indicated genotypes was PCR-amplified and the resulting amplification products are shown. (D) DRGs from mice of the indicated genotypes were dissected, dissociated, plated, and the resulting cells immunostained with the indicated primary antibodies. Representative micrographs of dissociated cells stained with the indicated antibodies are shown. Scale bar: 100 µm. (E) Quantification of the IRα staining intensity in dissociated Nav1.8^+^ DRG cells. Mean±s.e.m. *n*, number of cells quantified. Ordinary one-way ANOVA test followed by Tukey's multiple comparison test was used. **, *P*<0.01, ****, *P*<0.0001.

We next analyzed thermal and mechanical nociceptive sensitivity in the mice lacking *Insr* in Nav1.8^+^ nociceptive sensory neurons and relevant controls. Previously, it had been reported that mice with a broader deletion of *Insr* in sensory neurons using *Advillin-Cre* (Zurborg et al., 2011) had normal baselines for thermal and mechanical stimuli (Grote et al., 2018). Since flies lacking *InR* have normal baseline sensitivity but also exhibit persistent thermal (Im et al., 2018) and mechanical (Fig. 1) hypersensitivity following injury, we decided to test mice following a model of inflammatory pain induced by intraplantar injection of CFA (see Materials and Methods). Mice lacking *Insr* in Nav1.8^+^ neurons had normal thermal and mechanical sensitivity under baseline conditions, and no differences in the onset and resolution of thermal hyperalgesia following CFA injection into the hindpaw compared to controls (Fig. 3A). Male and female mice lacking *Insr* in Nav1.8^+^ neurons showed normal baseline nociception and a normal onset of mechanical hypersensitivity (Fig. 3B). However, in contrast with controls, the female mice showed a delayed resolution at later timepoints (days 14-21 following injury) (Fig. 3B). Male mice of both controls and lacking *Insr* in Nav1.8^+^ neurons were not distinguishable from controls (Fig. 3B). These data indicate that in an inflammatory pain assay mice lacking *Insr* in Nav1.8^+^ sensory neurons show a specific persistence of mechanical hypersensitivity only in females.

The observation of a sex-specific behavioral phenotype prompted us to revisit whether *Insr* was deleted properly in both male and female mice. PCR using primers capable of detecting a specific band following Cre-mediated deletion of the floxed exon 4 revealed apparently equivalent excision in both males and females (Fig. 3C). We further looked at Insr protein levels by immunostaining in dissociated short-term DRG cultures and observed a reduction of IRα staining in Nav1.8^+^ sensory neurons derived from both male and female mice (Fig. 3D,E). These results suggest that the deletion of *Insr* and the reduction of Insr protein is approximately equivalent in male and female mice.

### Metabolic activity in mice with a Nav1.8^+^ sensory neuron specific deletion of *Insr*

**Fig. 4.**
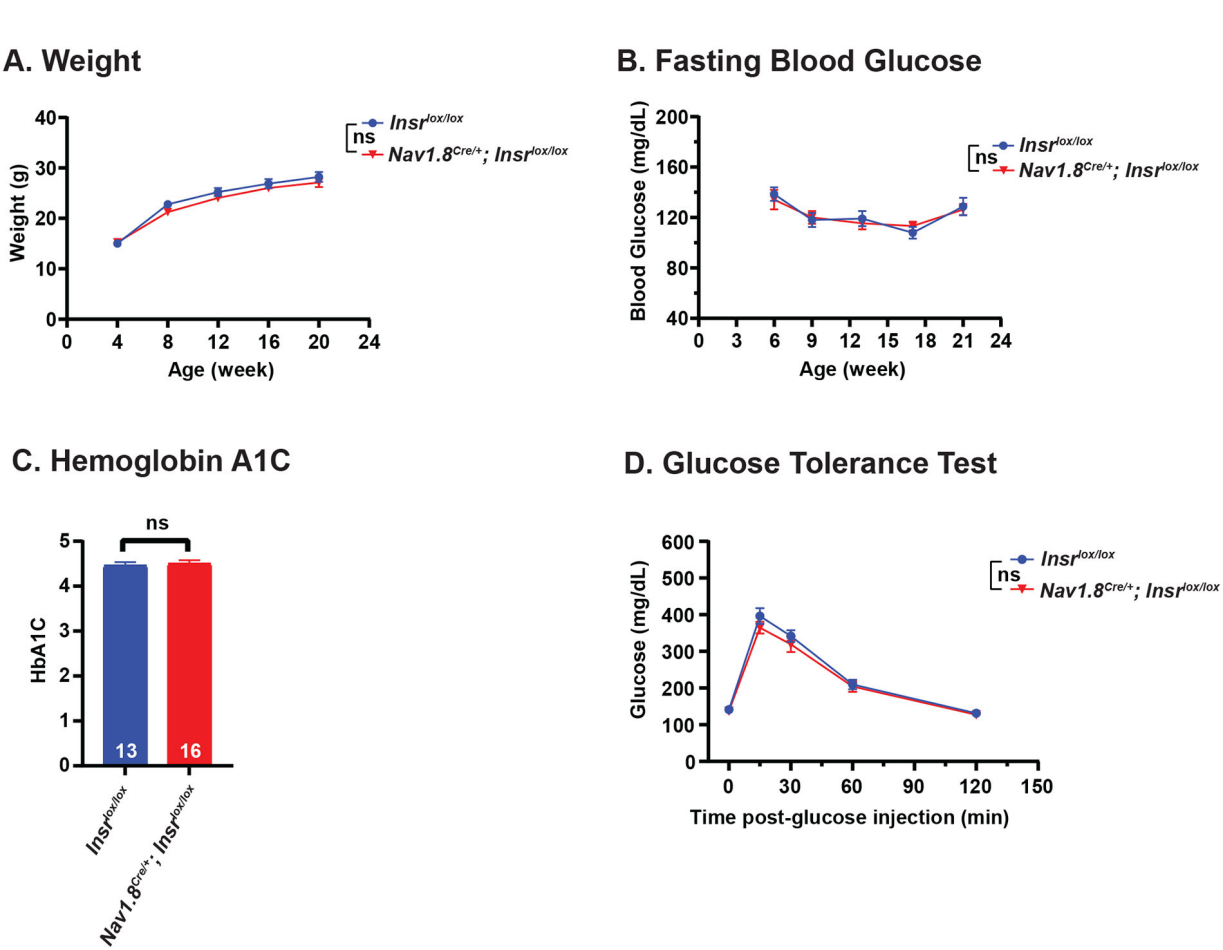
**Metabolic analysis of mice lacking sensory neuron-specific *Insr.*** (A) Body weight of *Nav1.8^Cre/+^;Insr^lox/lox^* mice and *Insr^lox/lox^* control mice as a function of the indicated ages. *Insr^lox/lox^*, *n*=22 (nine females and 13 males); *Nav1.8^Cre/+^;Insr^lox/lox^*, *n*=21 (15 females and six males). Because no sex-specific differences were observed in this and the other metabolic measures (B-D) the data are not separated by sex. Mean±s.e.m. Two-way repeated measures ANOVA with Geisser-Greenhouse correction followed by Sidak's multiple comparison test was used for statistical analysis. ns, not significant. (B) Blood glucose levels of *Nav1.8^Cre/+^;Insr^lox/lox^* and *Insr^lox/lox^* mice as a function of the indicated ages. *Insr^lox/lox^*, *n*=11 (five females and six males); *Nav1.8^Cre/+^;Insr^lox/lox^*, *n*=14 (eight females and six males). Mean±s.e.m. Two-way repeated measures ANOVA with Geisser-Greenhouse correction followed by Sidak's multiple comparison test was used for statistical analysis. ns, not significant. (C) Hemoglobin A1C levels of *Nav1.8^Cre/+^;Insr^lox/lox^* and *Insr^lox/lox^* mice. Hemoglobin A1C levels were measured at 21 weeks of age. *Insr^lox/lox^*, *n*=13 (six females and seven males); *Nav1.8^Cre/+^;Insr^lox/lox^*, *n*=16 (nine females and seven males). Mean±s.e.m. Two-tailed unpaired Student's *t*-test was used for statistical analysis. ns, not significant. (D) Glucose tolerance in *Nav1.8^Cre/+^;Insr^lox/lox^* and *Insr^lox/lox^* mice. An IPGTT was used to measure glucose levels immediately prior to glucose stimulation and at the indicated times thereafter. *Insr^lox/lox^*, *n*=13 (six females and seven males); *Nav1.8^Cre/+^;Insr^lox/lox^*, *n*=16 (nine females and seven males). Mean±s.e.m. Based on normality test, two-tailed unpaired Student's *t*-test was used except for 15 min for which two-tailed unpaired Mann–Whitney's test was used for statistical analysis. ns, not significant.

Although mouse sensory neurons are not generally thought to be a metabolic control tissue, we tested whether deletion of *Insr* in Nav1.8^+^ neurons had any systemic metabolic effects that might explain the observed persistent hypersensitivity in females. No sex-specific or other differences were observed between mutants and controls in body weight (Fig. 4A), fasting blood glucose (a measure of baseline blood glucose level at a single point in time) (Fig. 4B), Hemoglobin A1C levels (a measurement of average blood glucose levels over the past 2 to 3 months) (Fig. 4C), and glucose tolerance (a measurement of how quickly fasted mice move new sugar from the blood into the tissues) (Fig. 4D). The lack of difference between controls and mutant mice lacking *Insr* in Nav1.8^+^ sensory neurons suggests that the observed delay in resolution of mechanical hypersensitivity in female mice (Fig. 3B) cannot be explained by systemic metabolic effects.

## DISCUSSION

A prior study from our lab established that *Drosophila InR* regulates the persistence of injury-induced thermal nociceptive hypersensitivity (Im et al., 2018). We report here that *Drosophila* larvae mutant for *InR* or expressing *UAS-InR^RNAi^* transgenes in nociceptive sensory neurons exhibit normal baselines for mechanical nociception (at the low end of the dose-response curve) and normal acute sensitization. However, these larvae exhibit persistent mechanical allodynia and hyperalgesia following UV injury. This suggests that, in flies, *InR* regulation of the persistence of injury-induced nociceptive sensitization is not specific to heat sensitivity. This is perhaps not surprising since *Drosophila* class IV multidendritic (md) neurons are multimodal, mediating nociceptive responses both to high-threshold thermal stimuli (Babcock et al., 2009; Tracey et al., 2003) and mechanical stimuli (Hwang et al., 2007; Kim et al., 2012). The specific persistent hypersensitivity phenotype suggests that there is temporal regulation of InR activation following injury. Indeed, acute thermal hypersensitivity can be suppressed by nociceptor-specific activation of InR, whereas baseline sensitivity cannot (Im et al., 2018).

These *Drosophila* results prompted us to investigate whether *Insr* similarly regulates the persistence of inflammatory nociceptive sensitization in mice. We combined a floxed allele of *Insr* (Bruning et al., 1998) with a Cre driver specific for nociceptive sensory neurons (Agarwal et al., 2004) to delete *Insr* in these cells. As reported with a deletion of *Insr* in a broader subset of sensory neurons and in gut cells using *Advillin-Cre* (Zurborg et al., 2011), thermal and mechanical nociceptive baselines were normal in these mice (Grote et al., 2018). Here, since we also did not detect differences in baseline mechanical or thermal sensitivity, we further tested nociceptive sensitization following inflammatory injury. Baseline nociception for both sensory modalities was normal and acute thermal and mechanical hypersensitivity peaked normally in mice lacking *Insr* in Nav1.8^+^ sensory neurons versus controls. Two aspects of the response were different, compared to the results in *Drosophila*. First, no persistent thermal nociceptive hypersensitivity was observed. Second, the persistent mechanical nociceptive hypersensitivity was specific to female mice. The observation of mechanical hypersensitivity is consistent with reports that *Nav1.8-Cre* expresses in at least a subset of mechanically responsive sensory neurons (Daou et al., 2016; Shields et al., 2012). Several possibilities may explain the differences between flies and mice. First, the architecture of nociceptive sensory neurons in mice is more diverse, with a more clear physical and functional separation between neurons mediating thermal and mechanical responses (Jenkins and Lumpkin, 2017; Le Pichon and Chesler, 2014). Second, there have been reports of differing nociceptive sensitivity between male and female diabetic patients (Ruau et al., 2012). The sex-specific difference in behavior observed in this study is unlikely to be due to sex-specific differences in Cre-mediated excision of *Insr* exon 4 or reduction in Insr protein as these were observed to be similar. Whether there might be sex-specific differences in fly larvae is an interesting possibility for future testing, though we would note that at the larval stage *Drosophila* are not sexually active and lack mature genitalia. Similarly, whether the sex-specific difference in resolution of mechanical hypersensitivity is unique to the inflammatory injury paradigm employed here will be another promising area for further exploration.

One other study (Grote et al., 2018) has reported on sensory phenotypes of mice with a deletion of *Insr* in nociceptive sensory neurons. The driver used in this former study was *Advillin-Cre*, which expresses in a broader subset of sensory neurons but also expresses, like the *Advillin* promoter, in gut endocrine cells and enteric neurons (Hunter et al., 2018; Melo et al., 2020). In the Grote study, normal thermal and mechanical nociceptive baselines (without injury) were observed. They did not observe a difference in blood sugar levels but did observe differences in serum insulin levels and glucose tolerance that appeared in older mice (29+ weeks of age). Some of those same measures appeared normal in the experiments reported here. This raises the possibility that the metabolic effects observed previously were mediated either by sensory neurons not targeted by the *Nav1.8-Cre* driver used here or by enteric neurons or gut endocrine cells that express *Advillin*. We view the latter as more likely given that the gut is a known metabolic regulatory tissue (Drucker, 2007). Further experimentation will be needed to parse out these differences.

Finally, this study may have implications for the etiology of diabetes-associated pain (Obrosova, 2009). Some have argued that neuropathic pain in diabetic patients may have a predominantly central nervous system origin (Fischer and Waxman, 2010) whereas others have suggested possible roles for peripheral insulin signaling (Grote and Wright, 2016). In *Drosophila*, however, the persistent thermal nociceptive sensitization phenotype seen with sensory neuron specific knockdown of *InR* is precisely phenocopied in models of type 1 and type 2 diabetes (Im et al., 2018). Further, a recent study suggests that mechanical hypersensitivity is also observed in the type 2 diabetes model in fly larvae (Dabbara et al., 2021). In diabetic patients and experimental models, correlation of blood sugar control with pain hypersensitivity has been difficult to establish (Chan et al., 1990; Romanovsky et al., 2006). The potential role of regulated release of circulating insulin-like peptides in modulating nociceptive baselines has not been closely examined yet in either the fly or mouse model. Prominent models for diabetes-associated hypersensitivity include a secondary consequence of poor vascular tone (Kolka and Bergman, 2013; Powell et al., 1985) and possible adverse effects of glycation byproducts on sensory neurons (Matafome et al., 2017; Orestes et al., 2013). Our results suggest that another model is worth considering and testing further – that mechanical hypersensitivity may be related to poor insulin signaling directly within nociceptive sensory neurons, particularly in female vertebrates.

## MATERIALS AND METHODS

### Fly genetics

*Drosophila* were reared on standard cornmeal medium under a 12 h light-dark cycle. All crosses were cultured at 25°C except for the *InR* transheterozygous combination (*InR^e19/93Dj4^*) (Tatar et al., 2001), which was reared at 18°C until the third larval instar and then moved to 25°C for experiments. *w^1118^* and/or *Gal4^109(2)80^/+* (referred to as *md-Gal4,* a line that expresses in all four classes of peripheral multidendritic neurons in fly larvae) crossed to *w^1118^* served as control strains. *InR* mutant alleles used were a transheterozygous combination of *InR^e19^* and *InR^93Dj4^*. Both are loss of function measured by a decrease of kinase activity (Chen et al., 1996; Tatar et al., 2001). Tissue-specific expression of *UAS* transgenes was controlled by *md-Gal4* (Gao et al., 1999), which expresses in all four classes of multidendritic (md) sensory neurons. The *UAS-RNAi* lines (Ni et al., 2011) used was *InR^JF01482^*.

### Mouse genetics

All experimental protocols were approved by the Institutional Animal Care and Use Committee (IACUC) at The University of Texas MD Anderson Cancer Center and conformed to the U.S. National Institutes of Health guidelines on the ethical care and use of animals. Mice were housed 1-5 per cage and maintained on a 12 h light/dark schedule in a temperature-controlled environment with free access to food and reverse osmosis (RO) water.

*Scn10a^tm2(cre)Jwo^* (*Nav1.8^Cre/+^*) mice have been previously characterized, demonstrating sensory neuron specific Cre recombinase activity (Agarwal et al., 2004). *Insr^tm1Khn/^*^J^ (*Insr^lox/lox^*) mice with *loxP* sites flanking exon 4 of the insulin receptor gene (Bruning et al., 1998) were purchased from the Jackson Laboratory (JAX stock #006955, Bar Harbor, ME, USA). Cre-mediated deletion of exon 4 would create a nonfunctional 308 amino acid truncated protein. *Nav1.8^Cre/+^* mice were bred to *Insr^lox/lox^* to generate heterozygous *Nav1.8^Cre/+^; Insr^lox/+^* mice. *Nav1.8^Cre/+^; Insr^lox/+^* were bred to *Insr^lox/lox^* to produce mice with an *Nav1.8^Cre/+^; Insr^lox/lox^* genotype and littermate controls. These mice should have a specific deletion of *Insr* exon 4 within Nav1.8-expressing nociceptive sensory neurons. Mice were genotyped from DNA harvested from ear punches by Transnetyx (Cordova, TN, USA).

### *Drosophila* mechanical nociceptive sensitization assay

To create a UV-induced tissue injury (Babcock et al., 2009), mid L3 larvae anesthetized with anhydrous ethyl ether (Thermo Fisher Scientific, Waltham, MA, USA) were mounted on microscope slides so that the dorsal side was exposed to UV or mock-treated with 18-20 mJ cm^−2^ [measured with a UV spectrophotometer (Accumax XS-254; Spectronics Corporation, Melville, NY, USA)] in a Spectrolinker XL-1000 ultraviolet crosslinker (Spectronics Corporation, Melville, NY, USA). This manipulation leads to morphological disruption and apoptosis of a stripe of epidermal cells centered along the dorsal midline beginning about 20 h after injury (Babcock et al., 2009). The mechanical nociception assay for *Drosophila* larvae was performed as described in (Lopez-Bellido et al., 2019b). Briefly, custom made mechanical probes exerting mechanical pressures of 200 kPa (to measure mechanical allodynia) or 462 kPa (to measure mechanical hyperalgesia) were applied onto the posterior dorsal side of the larva at approximately abdominal segment A8 until the probe bends and exerts a constant pressure. The percentage of larvae that exhibited a nocifensive response – defined as a complete roll of 360° along the long axis of its body (within 2 s of probe bending) was recorded.

### Mouse nociception behavioral assays

#### Von Frey Test

Mice of 10-18 weeks old were acclimated to the mesh grid in individual plastic animal enclosures of 10×10×13 cm (1300 cm^3^) (IITC Life Sciences, Woodland Hills, CA, USA). The experimenter was blinded to the genotype of the tested mice. The up-down method (Dixon, 1980) was used to test mechanical sensitivity of the hindpaws. A set of von Frey monofilaments (Exacta Touch Test Sensory Evaluator, North Coast Medical, Gilroy, CA, USA) that exert forces of 0.008, 0.02, 0.04, 0.07, 0.16, 0.4, 0.6, 1, 1.4, and 2 g were applied to the plantar surface of the hind paw of each mouse, starting with the 0.4 g filament (Dixon, 1980). The duration of each stimulus was approximately 1 s and the inter-stimulus interval was approximately 30-60 s. The 50% withdrawal threshold for each hind paw was then calculated as described in (Chaplan et al., 1994) with a customized Python script. The 50% paw withdrawal threshold for each mouse was averaged from both hind paws. Baseline paw withdrawal threshold was averaged from measurement on three different days.

#### Hargreaves test

Mice of 10-13 weeks old were acclimated to the glass surface in individual plastic animal enclosures (IITC Life Sciences, Woodland Hills, CA, USA) as per an established protocol (Hargreaves et al., 1988) with modifications (Cheah et al., 2017). The experimenter was blinded to the genotype of the tested mice. The glass surface was maintained at 30°C. A focused radiant heat light beam of 30% intensity was chosen to elicit an average baseline withdrawal latency (sudden paw lifting from the glass) of ∼7 s and was aimed at the plantar surface of the hind paw and the paw withdrawal latency was recorded, repeating the procedure five times. The inter-test interval was at least 3 min. The average of three measurements after removing the minimal and maximal values (Cheah et al., 2017) was calculated for each hind paw with a customized Python script. The average paw withdrawal latency for each mouse was calculated from both hind paws. Baseline paw withdrawal latency was averaged from measurement on three different days prior to sensitization (below).

#### Inflammatory sensitization

Local inflammation in the paw was induced by intraplantar administration of Complete Freund's Adjuvant (CFA, 5 µl/hind paw; F5881, Sigma-Aldrich, St. Louis, MO, USA). The average paw withdrawal latency for mechanical and thermal nociception (see assays above) was measured on days 1, 3, 7, 10, 14 or 15, 21 after CFA administration.

### Mouse metabolism assays

Metabolic parameters for both control mice and mice with sensory-specific deletion of *Insr* were monitored in the afternoon at 6, 9, 13, 17, and 21 weeks of age. Mice weights were similarly measured at 4, 8, 12, 16, and 20 weeks of age.

#### Glucose and hemoglobin A1C

Blood glucose levels were determined using an Ascensia Contour Next Ez Blood Glucose Monitoring System (Parsippany, NJ, USA). Mice were fasted 3 h prior to glucose measurement and blood was collected via tail snip in the afternoon between 2 pm- 4 pm at 6, 9, 13, 17, and 21 weeks of age. In addition, long term glucose levels were assessed by determining hemoglobin A1C levels in the afternoon between 2 pm-4 pm at 21 weeks of age using A1CNow+ Professional Multi-Test HbA1c System (Pts Diagnostics, Indianapolis, IN, USA).

#### Intraperitoneal glucose tolerance test (IPGTT)

At 22 weeks of age, glucose tolerance was analyzed with an intraperitoneal glucose tolerance test (IPGTT) for both *Insr^lox/lox^* controls and *Nav1.8^Cre/+^;Insr^lox/lox^* experimental mice. After a 5-h fast, mice were administered 20% dextrose in saline at 10 ml kg^−1^ body weight via intraperitoneal injection. Blood glucose measurements were taken immediately prior to glucose administration and at 15, 30, 60 and 120 min thereafter in the afternoon between 3 pm-6 pm.

### *Insr* deletion and expression analysis

*PCR–*Genomic DNA was isolated from the dorsal root ganglion (DRG) and hindpaw skin of controls and *Nav1.8^Cre/+^;Insr^lox/lox^* mice to assess *Cre/loxP* recombination and deletion of *Insr* exon 4. The *Nav1.8-Cre* allele was detected using primers 5′- TGTAGATGGACTGCAGAGGATGGA-3′ and 5′-AAATGTTGCTGGATATTTTTACTGCC-3′ as described in (Gautron et al., 2011). The *Insr^lox/lox^* allele was detected using primers 5′-GATGTGCACCCCATGTCTG-3′ and 5′-CTGAATAGCTGAGACCACAG-3′ as described in (Bruning et al., 1998). PCR conditions were 94°C for 4 min followed by 30 cycles of 94°C for 40 s, 60°C for 30 s, 72°C for 25 s and a final extension at 72°C for 8 min. Cre/lox recombination and deletion of the exon 4 of the *Insr* gene was detected using primers 5′-ATGGTGGCATGCACTTATGA-3′ and 5′-TGCCTCAGCCTCCTGAATAG-3′. PCR conditions were 94°C for 4 min followed by 35 cycles of 94°C for 40 s, 54°C for 30 s, 72°C for 25 s and a final extension at 72°C for 8 min. In Fig. 3C, Cre/lox recombination and deletion of the exon 4 of the *Insr* gene was detected using primers 5′-CAACCGTGCCTAGAGACTCC-3′ and 5′-CTGAATAGCTGAGACCACAG-3′. PCR conditions were 94°C for 4 min followed by 35 cycles of 94°C for 40 s, 58°C for 20 s, 72°C for 20 s and a final extension at 72°C for 8 min.

### Immunofluorescence, microscopy and quantification of IRα in DRG sections

DRGs were fixed in 4% paraformaldehyde overnight at 4°C followed by cryoprotection in 30% sucrose overnight at 4°C. Fixed samples were embedded in O.C.T (Thermo Fisher Scientific, Waltham, MA, USA) for cryosectioning at 10 µm with a Cryostar NX70 cryostat (Thermo Fisher Scientific, Waltham, MA, USA). Collected sections were mounted on glass slides, blocked in blocking buffer (5% goat serum, 0.3% Triton X-100 in PBS) (all materials here from Thermo Fisher Scientific, Waltham, MA, USA, except goat serum- G9023, Sigma-Aldrich, St. Louis, MO, USA) for 1 h, and stained with primary antibodies overnight at 4°C. The primary antibodies were guinea pig anti-Nav1.8 [1:100, AGP-029 (currently ASC-016-GP), Alomone Labs, Jerusalem, Israel], chicken-anti-GFAP (1:100, PA1-10004, Thermo Fisher Scientific, Waltham, MA, USA), and rabbit anti-IRα (1:25, ab5500, Abcam, Cambridge, MA, USA). Dilutions were in antibody dilution buffer (1% BSA, 0.3% Triton X-100 in PBS- all materials here from Thermo Fisher Scientific, Waltham, MA, USA). After washing with wash buffer (0.05% Tween-20 in PBS), the samples were incubated in secondary antibodies for 1 h. The secondary antibodies were goat anti-guinea pig Alexa Fluro Cy3 (1:25, 106-165-003, Jackson ImmunoResearch, West Grove, PA, USA), goat anti-chicken Alexa Fluor 488 (1:100; A11039, Thermo Fisher Scientific, Waltham, MA, USA), and goat anti-rabbit Alexa Fluor 647 (1:100, ab150083, Abcam, Cambridge, MA, USA). After washing, the sections were covered in Vectashield (H-1000, Vector Laboratories, Burlingame, CA, USA) for imaging. Images were captured with a Leica MZ16 FA fluorescent stereomicroscope equipped with a PLAN APO 1.6x stereo-objective and a HAMAMATSU ORCA-ER C-4742-80-12AG digital camera using Leica LAS X. The mean intensity of IRα in Nav1.8^+^ and GFAP^+^ DRG region was measured using ImageJ. Nav1.8^+^ and GFAP^+^ region was identified using threshold method with same settings for all images.

### Immunofluorescence, microscopy and quantification of IRα in dissociated DRG cells

DRGs were incubated in DMEM/F12 (10-090-CV, Thermo Fisher Scientific, Waltham, MA, USA) containing 10 mM HEPES (MT25060CI, Thermo Fisher Scientific, Waltham, MA, USA), 10% fetal bovine serum (16000044, Thermo Fisher Scientific, Waltham, MA, USA), 1.5 mg ml^−1^ collagenase Type IA (C9891, Sigma-Aldrich, St. Louis, MO, USA) and 200 µg ml^−1^ DNase I (D5025-15KU, Sigma-Aldrich, St. Louis, MO, USA) for 20 min at 37°C, followed by trituration with P-1000 pipet tip and then P-200 pipet tip until they completely dissociated. After cell dispersion in Petri dishes, loosely attached DRG cells were collected, filtered through a 70 µm cell strainer (352350, Thermo Fisher Scientific, Waltham, MA, USA) and plated on collagen Type I (08-115, Millipore Sigma, Burlington, MA, USA)-coated Millicell EZ slide (PEZGS0816, Millipore Sigma, Burlington, MA, USA) in DMEM/F12 containing 10 mM HEPES, 10% fetal bovine serum for 10 min. Cells were fixed in 3.7% formaldehyde for 20 min, permeabilized with 0.3% triton X-100 for 5 min, and blocked in PBS containing 10% heat-inactivated goat serum for 1 h before immunostaining. The primary antibodies were guinea pig anti-Nav1.8 (1:100, ASC-016-GP, Alomone Labs, Jerusalem, Israel), and rabbit anti-IRα (1:25, ab5500 Abcam, Cambridge, MA, USA) in PBS containing 1% BSA, 0.1% Tween 20 for 2 h. The secondary antibodies were goat anti-guinea pig Alexa Fluro 488 (1:200, 106-545-003, Jackson ImmunoResearch, West Grove, PA, USA), goat anti-rabbit Alexa Fluor 647 (1:200, A21244, Thermo Fisher Scientific, Waltham, MA, USA) for 1 h. After washing, the cells were covered in Vectashield (H-1000, Vector Laboratories, Burlingame, CA, USA) for imaging. Images were captured with a Nikon confocal microscope and a 20X 0.75NA objective using NIS-Elements AR 5.30.05 software. The mean intensity of IRα in Nav1.8^+^ DRG cells was measured using ImageJ.

### Statistical analyses

#### Mouse data

All data is reported as the mean±standard error of the mean. All the data were tested for a normal distribution using either Kolmogorov–Smirnov (KS), D'Agostino & Pearson or Shapiro–Wilk normality test. For comparison of two groups of data that passed normality test, two-tailed unpaired Student's *t*-test was used; otherwise, two-tailed unpaired Mann–Whitney test was used. For comparison of more than two groups of data that did not involve time and passed normality test, ordinary one-way ANOVA test followed by Tukey's multiple comparison test was used. For data involving time and all timepoint data passed normality test, repeated measures two-way ANOVA test with Geisser-Greenhouse correction followed by Sidak's multiple comparison test was used. For data that not all timepoint data passed normality test, each timepoint data was separately analyzed using two-tailed unpaired Student's *t*-test or two-tailed unpaired Mann–Whitney test based on normality test result. Graphpad Prism software (GraphPad Software, La Jolla, CA, USA) was used for all statistical analyses (including for the fly data, as described below).

#### Fly behavioral data

All the data were tested for a normal distribution using KS or Shapiro–Wilk normality test. To compare two groups, two-tailed unpaired Student's *t*-test was used. To compare more than two groups, a one-way ANOVA (followed by Tukey post hoc test) was used.

## References

[BIO059864C1] Accili, D., Drago, J., Lee, E. J., Johnson, M. D., Cool, M. H., Salvatore, P., Asico, L. D., José, P. A., Taylor, S. I. and Westphal, H. (1996). Early neonatal death in mice homozygous for a null allele of the insulin receptor gene. *Nat. Genet.* 12, 106-109. 10.1038/ng0196-106 8528241

[BIO059864C2] Agarwal, N., Offermanns, S. and Kuner, R. (2004). Conditional gene deletion in primary nociceptive neurons of trigeminal ganglia and dorsal root ganglia. *Genesis* 38, 122-129. 10.1002/gene.20010 15048809

[BIO059864C3] Babcock, D. T., Landry, C. and Galko, M. J. (2009). Cytokine signaling mediates UV-induced nociceptive sensitization in *Drosophila* larvae. *Curr. Biol.* 19, 799-806. 10.1016/j.cub.2009.03.062 19375319 PMC4017352

[BIO059864C4] Babcock, D. T., Shi, S., Jo, J., Shaw, M., Gutstein, H. B. and Galko, M. J. (2011). Hedgehog signaling regulates nociceptive sensitization. *Curr. Biol.* 21, 1525-1533. 10.1016/j.cub.2011.08.020 21906949 PMC3262399

[BIO059864C5] Böhni, R., Riesgo-Escovar, J., Oldham, S., Brogiolo, W., Stocker, H., Andruss, B. F., Beckingham, K. and Hafen, E. (1999). Autonomous control of cell and organ size by CHICO, a *Drosophila* homolog of vertebrate IRS1-4. *Cell* 97, 865-875. 10.1016/S0092-8674(00)80799-0 10399915

[BIO059864C6] Bruning, J. C., Michael, M. D., Winnay, J. N., Hayashi, T., Horsch, D., Accili, D., Goodyear, L. J. and Kahn, C. R. (1998). A muscle-specific insulin receptor knockout exhibits features of the metabolic syndrome of NIDDM without altering glucose tolerance. *Mol. Cell* 2, 559-569. 10.1016/S1097-2765(00)80155-0 9844629

[BIO059864C7] Brüning, J. C., Gautam, D., Burks, D. J., Gillette, J., Schubert, M., Orban, P. C., Klein, R., Krone, W., Müller-Wieland, D. and Kahn, C. R. (2000). Role of brain insulin receptor in control of body weight and reproduction. *Science* 289, 2122-2125. 10.1126/science.289.5487.2122 11000114

[BIO059864C8] Chan, A. W., Macfarlane, I. A. and Bowsher, D. (1990). Short term fluctuations in blood glucose concentrations do not alter pain perception in diabetic-patients with and without painful peripheral neuropathy. *Diabetes Res.* 14, 15-19. 2134661

[BIO059864C9] Chaplan, S. R., Bach, F. W., Pogrel, J. W., Chung, J. M. and Yaksh, T. L. (1994). Quantitative assessment of tactile allodynia in the rat paw. *J. Neurosci. Methods* 53, 55-63. 10.1016/0165-0270(94)90144-9 7990513

[BIO059864C10] Cheah, M., Fawcett, J. W. and Andrews, M. R. (2017). Assessment of thermal pain sensation in rats and mice using the Hargreaves test. *Bio. Protoc.* 7, e2506. 10.21769/BioProtoc.2506 PMC5600253 28920069

[BIO059864C11] Chen, C., Jack, J. and Garofalo, R. S. (1996). The *Drosophila* insulin receptor is required for normal growth. *Endocrinology* 137, 846-856. 10.1210/endo.137.3.8603594 8603594

[BIO059864C12] Clancy, D. J., Gems, D., Harshman, L. G., Oldham, S., Stocker, H., Hafen, E., Leevers, S. J. and Partridge, L. (2001). Extension of life-span by loss of CHICO, a *Drosophila* insulin receptor substrate protein. *Science* 292, 104-106. 10.1126/science.1057991 11292874

[BIO059864C13] Cunha, F. Q., Poole, S., Lorenzetti, B. B. and Ferreira, S. H. (1992). The pivotal role of tumour necrosis factor alpha in the development of inflammatory hyperalgesia. *Br. J. Pharmacol.* 107, 660-664. 10.1111/j.1476-5381.1992.tb14503.x 1472964 PMC1907751

[BIO059864C14] Cunha, T. M., Verri, W. A., Jr., Silva, J. S., Poole, S., Cunha, F. Q. and Ferreira, S. H. (2005). A cascade of cytokines mediates mechanical inflammatory hypernociception in mice. *Proc. Natl. Acad. Sci. USA* 102, 1755-1760. 10.1073/pnas.0409225102 15665080 PMC547882

[BIO059864C15] Dabbara, H., Schultz, A. and Im, S. H. (2021). *Drosophila* insulin receptor regulates diabetes-induced mechanical nociceptive hypersensitivity. *MicroPubl. Biol.* 2021. 10.17912/micropub.biology.000456 PMC8449261 34549177

[BIO059864C16] Daou, I., Beaudry, H., Ase, A. R., Wieskopf, J. S., Ribeiro-Da-Silva, A., Mogil, J. S. and Séguéla, P. (2016). Optogenetic silencing of Nav1.8-positive afferents alleviates inflammatory and neuropathic pain. *eNeuro* 3, ENEURO.0140-15.2016. 10.1523/ENEURO.0140-15.2016 PMC4794527 27022626

[BIO059864C17] Dixon, W. J. (1980). Efficient analysis of experimental observations. *Annu. Rev. Pharmacol. Toxicol.* 20, 441-462. 10.1146/annurev.pa.20.040180.002301 7387124

[BIO059864C18] Drucker, D. J. (2007). The role of gut hormones in glucose homeostasis. *J. Clin. Invest.* 117, 24-32. 10.1172/JCI30076 17200703 PMC1716213

[BIO059864C19] Fernandez, R., Tabarini, D., Azpiazu, N., Frasch, M. and Schlessinger, J. (1995). The *Drosophila* insulin receptor homolog: a gene essential for embryonic development encodes two receptor isoforms with different signaling potential. *EMBO J.* 14, 3373-3384. 10.1002/j.1460-2075.1995.tb07343.x 7628438 PMC394404

[BIO059864C20] Fischer, T. Z. and Waxman, S. G. (2010). Neuropathic pain in diabetes--evidence for a central mechanism. *Nat. Rev. Neurol.* 6, 462-466. 10.1038/nrneurol.2010.90 20625378

[BIO059864C21] Gao, F. B., Brenman, J. E., Jan, L. Y. and Jan, Y. N. (1999). Genes regulating dendritic outgrowth, branching, and routing in *Drosophila*. *Genes Dev.* 13, 2549-2561. 10.1101/gad.13.19.2549 10521399 PMC317067

[BIO059864C22] Gautron, L., Sakata, I., Udit, S., Zigman, J. M., Wood, J. N. and Elmquist, J. K. (2011). Genetic tracing of Nav1.8-expressing vagal afferents in the mouse. *J. Comp. Neurol.* 519, 3085-3101. 10.1002/cne.22667 21618224 PMC3306808

[BIO059864C23] Goberdhan, D. C., Paricio, N., Goodman, E. C., Mlodzik, M. and Wilson, C. (1999). *Drosophila* tumor suppressor PTEN controls cell size and number by antagonizing the Chico/PI3-kinase signaling pathway. *Genes Dev.* 13, 3244-3258. 10.1101/gad.13.24.3244 10617573 PMC317204

[BIO059864C24] Gold, M. S. and Gebhart, G. F. (2010). Nociceptor sensitization in pain pathogenesis. *Nat. Med.* 16, 1248-1257. 10.1038/nm.2235 20948530 PMC5022111

[BIO059864C25] Grote, C. W. and Wright, D. E. (2016). A role for insulin in diabetic neuropathy. *Front. Neurosci.* 10, 581. 10.3389/fnins.2016.00581 28066166 PMC5179551

[BIO059864C26] Grote, C. W., Wilson, N. M., Katz, N. K., Guilford, B. L., Ryals, J. M., Novikova, L., Stehno-Bittel, L. and Wright, D. E. (2018). Deletion of the insulin receptor in sensory neurons increases pancreatic insulin levels. *Exp. Neurol.* 305, 97-107. 10.1016/j.expneurol.2018.04.002 29649429 PMC5963702

[BIO059864C27] Hargreaves, K., Dubner, R., Brown, F., Flores, C. and Joris, J. (1988). A new and sensitive method for measuring thermal nociception in cutaneous hyperalgesia. *Pain* 32, 77-88. 10.1016/0304-3959(88)90026-7 3340425

[BIO059864C28] Hu, C., Petersen, M., Hoyer, N., Spitzweck, B., Tenedini, F., Wang, D., Gruschka, A., Burchardt, L. S., Szpotowicz, E., Schweizer, M. et al. (2017). Sensory integration and neuromodulatory feedback facilitate *Drosophila* mechanonociceptive behavior. *Nat. Neurosci.* 20, 1085-1095. 10.1038/nn.4580 28604684 PMC5931224

[BIO059864C29] Hunter, D. V., Smaila, B. D., Lopes, D. M., Takatoh, J., Denk, F. and Ramer, M. S. (2018). advillin is expressed in all adult neural crest-derived neurons. *eNeuro* 5, ENEURO.0077-18.2018. 10.1523/ENEURO.0077-18.2018 PMC6135988 30221190

[BIO059864C30] Hwang, R. Y., Zhong, L., Xu, Y., Johnson, T., Zhang, F., Deisseroth, K. and Tracey, W. D. (2007). Nociceptive neurons protect *Drosophila* larvae from parasitoid wasps. *Curr. Biol.* 17, 2105-2116. 10.1016/j.cub.2007.11.029 18060782 PMC2225350

[BIO059864C31] Hwang, R. Y., Stearns, N. A. and Tracey, W. D. (2012). The ankyrin repeat domain of the TRPA protein painless is important for thermal nociception but not mechanical nociception. *PLoS One* 7, e30090. 10.1371/journal.pone.0030090 22295071 PMC3266251

[BIO059864C32] Im, S. H. and Galko, M. J. (2012). Pokes, sunburn, and hot sauce: *Drosophila* as an emerging model for the biology of nociception. *Dev. Dyn.* 241, 16-26. 10.1002/dvdy.22737 21932321 PMC3258975

[BIO059864C33] Im, S. H., Takle, K., Jo, J., Babcock, D. T., Ma, Z., Xiang, Y. and Galko, M. J. (2015). Tachykinin acts upstream of autocrine Hedgehog signaling during nociceptive sensitization in *Drosophila*. *Elife* 4, e10735. 10.7554/eLife.10735 26575288 PMC4739760

[BIO059864C34] Im, S. H., Patel, A. A., Cox, D. N. and Galko, M. J. (2018). *Drosophila* Insulin receptor regulates the persistence of injury-induced nociceptive sensitization. *Dis. Model. Mech.* 11, dmm034231. 10.1242/dmm.034231 29752280 PMC5992604

[BIO059864C35] Jenkins, B. A. and Lumpkin, E. A. (2017). Developing a sense of touch. *Development* 144, 4078-4090. 10.1242/dev.120402 29138290 PMC5719243

[BIO059864C36] Kang, K., Pulver, S. R., Panzano, V. C., Chang, E. C., Griffith, L. C., Theobald, D. L. and Garrity, P. A. (2010). Analysis of *Drosophila* TRPA1 reveals an ancient origin for human chemical nociception. *Nature* 464, 597-600. 10.1038/nature08848 20237474 PMC2845738

[BIO059864C37] Kim, S. E., Coste, B., Chadha, A., Cook, B. and Patapoutian, A. (2012). The role of *Drosophila* Piezo in mechanical nociception. *Nature* 483, 209-212. 10.1038/nature10801 22343891 PMC3297676

[BIO059864C38] Kolka, C. M. and Bergman, R. N. (2013). The endothelium in diabetes: its role in insulin access and diabetic complications. *Rev. Endocr. Metab. Disord.* 14, 13-19. 10.1007/s11154-012-9233-5 23306780 PMC3593803

[BIO059864C39] Laird, J. M., Roza, C., De Felipe, C., Hunt, S. P. and Cervero, F. (2001). Role of central and peripheral tachykinin NK1 receptors in capsaicin-induced pain and hyperalgesia in mice. *Pain* 90, 97-103. 10.1016/S0304-3959(00)00394-8 11166975

[BIO059864C40] Le Pichon, C. E. and Chesler, A. T. (2014). The functional and anatomical dissection of somatosensory subpopulations using mouse genetics. *Front. Neuroanat.* 8, 21. 10.3389/fnana.2014.00021 24795573 PMC4001001

[BIO059864C41] Liu, S., Lv, Y., Wan, X. X., Song, Z. J., Liu, Y. P., Miao, S., Wang, G. L. and Liu, G. J. (2018). Hedgehog signaling contributes to bone cancer pain by regulating sensory neuron excitability in rats. *Mol. Pain* 14, 1744806918767560. 10.1177/1744806918767560 29607715 PMC5888817

[BIO059864C42] Lopez-Bellido, R. and Galko, M. J. (2020). An improved assay and tools for measuring mechanical nociception in *Drosophila* larvae. *J. Vis. Exp*. 164, 10.3791/61911 PMC7899084 33191934

[BIO059864C43] Lopez-Bellido, R., Himmel, N. J., Gutstein, H. B., Cox, D. N. and Galko, M. J. (2019a). An assay for chemical nociception in *Drosophila* larvae. *Philos. Trans. R. Soc. Lond. B Biol. Sci.* 374, 20190282. 10.1098/rstb.2019.0282 31544619 PMC6790381

[BIO059864C44] Lopez-Bellido, R., Puig, S., Huang, P. J., Tsai, C. R., Turner, H. N., Galko, M. J. and Gutstein, H. B. (2019b). Growth factor signaling regulates mechanical nociception in flies and vertebrates. *J. Neurosci.* 39, 6012-6030. 10.1523/JNEUROSCI.2950-18.2019 31138657 PMC6650988

[BIO059864C45] Matafome, P., Rodrigues, T., Sena, C. and Seiça, R. (2017). Methylglyoxal in metabolic disorders: facts, myths, and promises. *Med. Res. Rev.* 37, 368-403. 10.1002/med.21410 27636890

[BIO059864C46] Mauthner, S. E., Hwang, R. Y., Lewis, A. H., Xiao, Q., Tsubouchi, A., Wang, Y., Honjo, K., Skene, J. H., Grandl, J. and Tracey, W. D. Jr. (2014). Balboa binds to pickpocket in vivo and is required for mechanical nociception in *Drosophila* larvae. *Curr. Biol.* 24, 2920-2925. 10.1016/j.cub.2014.10.038 25454784 PMC4438766

[BIO059864C47] Melo, C. G. S., Nicolai, E. N., Alcaino, C., Cassmann, T. J., Whiteman, S. T., Wright, A. M., Miller, K. E., Gibbons, S. J., Beyder, A. and Linden, D. R. (2020). Identification of intrinsic primary afferent neurons in mouse jejunum. *Neurogastroenterol. Motil.* 32, e13989. 10.1111/nmo.13989 32986284 PMC8114175

[BIO059864C48] Milinkeviciute, G., Gentile, C. and Neely, G. G. (2012). *Drosophila* as a tool for studying the conserved genetics of pain. *Clin. Genet.* 82, 359-366. 10.1111/j.1399-0004.2012.01941.x 22880632

[BIO059864C49] Musselman, L. P., Fink, J. L., Narzinski, K., Ramachandran, P. V., Hathiramani, S. S., Cagan, R. L. and Baranski, T. J. (2011). A high-sugar diet produces obesity and insulin resistance in wild-type *Drosophila*. *Dis. Model. Mech.* 4, 842-849. 10.1242/dmm.007948 21719444 PMC3209653

[BIO059864C50] Ni, J. Q., Zhou, R., Czech, B., Liu, L. P., Holderbaum, L., Yang-Zhou, D., Shim, H. S., Tao, R., Handler, D., Karpowicz, P. et al. (2011). A genome-scale shRNA resource for transgenic RNAi in *Drosophila*. *Nat. Methods* 8, 405-407. 10.1038/nmeth.1592 21460824 PMC3489273

[BIO059864C51] Obrosova, I. G. (2009). Diabetic painful and insensate neuropathy: pathogenesis and potential treatments. *Neurotherapeutics* 6, 638-647. 10.1016/j.nurt.2009.07.004 19789069 PMC5084286

[BIO059864C52] Ohyama, T., Schneider-Mizell, C. M., Fetter, R. D., Aleman, J. V., Franconville, R., Rivera-Alba, M., Mensh, B. D., Branson, K. M., Simpson, J. H., Truman, J. W. et al. (2015). A multilevel multimodal circuit enhances action selection in *Drosophila*. *Nature* 520, 633-639. 10.1038/nature14297 25896325

[BIO059864C53] Orestes, P., Osuru, H. P., Mcintire, W. E., Jacus, M. O., Salajegheh, R., Jagodic, M. M., Choe, W., Lee, J., Lee, S. S., Rose, K. E. et al. (2013). Reversal of neuropathic pain in diabetes by targeting glycosylation of Ca(V)3.2 T-type calcium channels. *Diabetes* 62, 3828-3838. 10.2337/db13-0813 23835327 PMC3806612

[BIO059864C54] Powell, H. C., Rosoff, J. and Myers, R. R. (1985). Microangiopathy in human diabetic neuropathy. *Acta Neuropathol.* 68, 295-305. 10.1007/BF00690832 4090941

[BIO059864C55] Romanovsky, D., Cruz, N. F., Dienel, G. A. and Dobretsov, M. (2006). Mechanical hyperalgesia correlates with insulin deficiency in normoglycemic streptozotocin-treated rats. *Neurobiol. Dis.* 24, 384-394. 10.1016/j.nbd.2006.07.009 16935517

[BIO059864C56] Ruau, D., Liu, L. Y., Clark, J. D., Angst, M. S. and Butte, A. J. (2012). Sex differences in reported pain across 11,000 patients captured in electronic medical records. *J. Pain* 13, 228-234. 10.1016/j.jpain.2011.11.002 22245360 PMC3293998

[BIO059864C57] Rulifson, E. J., Kim, S. K. and Nusse, R. (2002). Ablation of insulin-producing neurons in flies: growth and diabetic phenotypes. *Science* 296, 1118-1120. 10.1126/science.1070058 12004130

[BIO059864C58] Shields, S. D., Ahn, H. S., Yang, Y., Han, C., Seal, R. P., Wood, J. N., Waxman, S. G. and Dib-Hajj, S. D. (2012). Nav1.8 expression is not restricted to nociceptors in mouse peripheral nervous system. *Pain* 153, 2017-2030. 10.1016/j.pain.2012.04.022 22703890

[BIO059864C59] Sugimoto, K., Murakawa, Y., Zhang, W., Xu, G. and Sima, A. A. (2000). Insulin receptor in rat peripheral nerve: its localization and alternatively spliced isoforms. *Diabetes Metab. Res. Rev.* 16, 354-363. 10.1002/1520-7560(200009/10)16:5<354::AID-DMRR149>3.0.CO;2-H 11025559

[BIO059864C60] Sugimoto, K., Murakawa, Y. and Sima, A. A. (2002). Expression and localization of insulin receptor in rat dorsal root ganglion and spinal cord. *J. Peripher. Nerv. Syst.* 7, 44-53. 10.1046/j.1529-8027.2002.02005.x 11939351

[BIO059864C61] Tatar, M., Kopelman, A., Epstein, D., Tu, M.-P., Yin, C.-M. and Garofalo, R. S. (2001). A mutant *Drosophila* insulin receptor homolog that extends life-span and impairs neuroendocrine function. *Science* 292, 107-110. 10.1126/science.1057987 11292875

[BIO059864C62] Tracey, W. D., Jr., Wilson, R. I., Laurent, G. and Benzer, S. (2003). painless, a *Drosophila* gene essential for nociception. *Cell* 113, 261-273. 10.1016/S0092-8674(03)00272-1 12705873

[BIO059864C63] Turner, H. N., Armengol, K., Patel, A. A., Himmel, N. J., Sullivan, L., Iyer, S. C., Bhattacharya, S., Iyer, E. P. R., Landry, C., Galko, M. J. et al. (2016). The TRP channels Pkd2, NompC, and Trpm act in cold-sensing neurons to mediate unique aversive behaviors to Noxious cold in *Drosophila*. *Curr. Biol.* 26, 3116-3128. 10.1016/j.cub.2016.09.038 27818173 PMC5140760

[BIO059864C64] Weng, H. R., Mansikka, H., Winchurch, R., Raja, S. N. and Dougherty, P. M. (2001). Sensory processing in the deep spinal dorsal horn of neurokinin-1 receptor knockout mice. *Anesthesiology* 94, 1105-1112. 10.1097/00000542-200106000-00027 11465604

[BIO059864C65] Yoshino, J., Morikawa, R. K., Hasegawa, E. and Emoto, K. (2017). Neural circuitry that evokes escape behavior upon activation of nociceptive sensory neurons in *Drosophila* Larvae. *Curr. Biol.* 27, 2499-2504.e3. 10.1016/j.cub.2017.06.068 28803873

[BIO059864C66] Zurborg, S., Piszczek, A., Martínez, C., Hublitz, P., Al Banchaabouchi, M., Moreira, P., Perlas, E. and Heppenstall, P. A. (2011). Generation and characterization of an Advillin-Cre driver mouse line. *Mol. Pain* 7, 66. 10.1186/1744-8069-7-66 21906401 PMC3185264

